# Experimental Research Regarding the Effect of Mineral Aggregates on the Wear of Mixing Blades of Concrete Mixers

**DOI:** 10.3390/ma16145047

**Published:** 2023-07-17

**Authors:** Adrian Niță, Eugen Laudacescu, Marius Gabriel Petrescu, Teodor Dumitru, Andrei Burlacu, Dorin George Bădoiu, Maria Tănase

**Affiliations:** Mechanical Engineering Department, Petroleum–Gas University of Ploiești, 100680 Ploiești, Romania; adrian.nita@upg-ploiesti.ro (A.N.); pmarius@upg-ploiesti.ro (M.G.P.); teodor.dumitru@upg-ploiesti.ro (T.D.); andrei-ion@upg-ploiesti.ro (A.B.); dorin.badoiu@gmail.com (D.G.B.); maria.tanase@upg-ploiesti.ro (M.T.)

**Keywords:** concrete mixer, aggregate, experimental stand, blades, cast iron, angle of attack

## Abstract

Industrial mixers are equipment used to mechanically combine different types of materials in order to obtain homogeneous mixtures. In concrete industry production, mixers play a crucial role by facilitating the efficient and consistent blending of various constituents to create high-quality concrete. Because the mixers in the concrete industry work in conditions characterized by abrasive and erosive loadings, the authors of this paper tried to establish a dependence between the quality of the material from which the mixing elements are made and their wear resistance. Three types of cast irons alloyed with chromium, specific to the construction of mixing blades, were used in this research. The working environment was a mixture of crushed mineral aggregates corresponding to the granulometric class 4–8 mm. The tests were carried out on an experimental stand designed and built by the authors of this paper. The stand reproduces on a scale of 1:2 a drum made up of a double-axis horizontal mixer. The stand had the possibility to change the value of the attack angle of the mixing blades, corresponding to the following values: 30, 45, and 60 degrees. The results of the tests established the dependence between the type of material and the wear rate of the blades as well as the influence exerted by the angle of attack on the wear of the mixing blades. It was shown that when the inclination angle of the blade relative to the shaft axis increases, the cumulative mass loss decreases, with values between 43% and 55.83%, as a function of the quality of blade material.

## 1. Introduction

The loadings of the mixing elements of the mixer but also of its tank armor are determined by the complex phenomena of abrasive–erosive–corrosive wear due to very aggressive simultaneous actions determined by the friction of the mineral aggregates on the active surfaces of the mixer, the impact of the aggregate particles on the active surfaces of the mixer, and the corrosive action of cement in combination with water [1,2,3,4,5,6].

**Figure 1:**
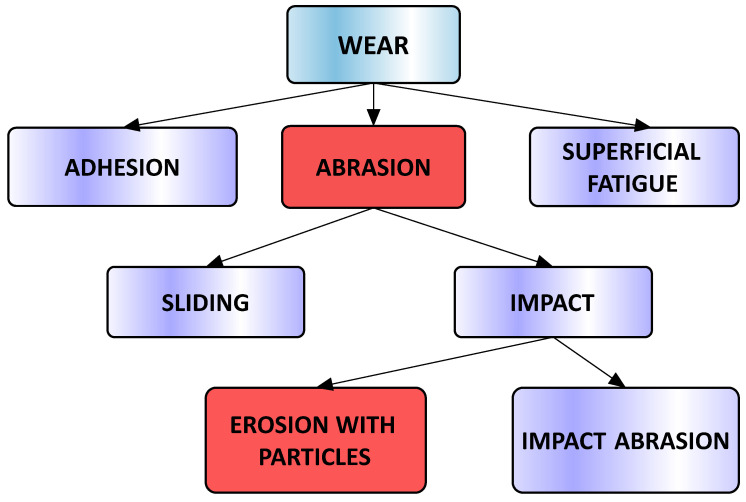
Wear phenomena specific to concrete mixers.

The wear of the component elements of concrete mixers and the removal of material due to this phenomenon can manifest in various forms, such as adhesion, abrasion, and surface fatigue (Figure 1).

According to the results from the scientific papers [7,8,9,10], the level of mechanical stress in mixers is strongly influenced by factors such as the angle of impact, the impact velocity, and the hardness of the wet components within the mixer.

The article [7] provides an extensive examination of the key factors influencing wear on impellers in solid–liquid slurry applications, as well as strategies for minimizing the wear. The critical parameters explored include the following aspects: the chemical environment, hardness of solids, density of solids, percent solids, shape of solids, fluid regime (ranging from turbulent to transitional or laminar), hardness of the mixer’s wetted parts, hydraulic efficiency of the impeller (including kinetic energy dissipation rates near the impeller blades), impact velocity, and impact frequency.

Sapate [8] examined the impact of erodent-particle hardness on the velocity exponent of certain weld deposited alloys and focused on three steels and two alloy cast irons, with bulk hardness ranging from 300 to 800 HV. The erodent particles used in the tests had hardness values ranging from 400 to 1875 HV. Erosion tests were conducted using cement clinker, blast-furnace sinter, silica sand, and alumina particles with sizes ranging from 125 to 150 µm. The impingement angles tested were 30° and 90°, and the impingement velocities varied from 25 to 120 m/sec. Regardless of the erodent-particle hardness and impingement angle, an increasing trend was observed between the velocity exponent and the hardness of the alloys. Similarly, the velocity exponent increased with higher erodent-particle hardness, independent of the impingement angle and alloy hardness.

The main findings of [9] indicate that velocity exponents derived for “erosion-dominated” conditions are similar to those observed for “ductile” erosive processes. However, in “corrosion-dominated” conditions, the velocity exponents are significantly lower compared to those found in “brittle” erosive processes at room temperature. In cases of “erosion–corrosion-dominated” conditions, the velocity exponents exhibit strong dependence on temperature, alloy composition, and the relationship between velocity and particle flux.

The objective of study [10] was to design and construct a wear-testing rig specifically for a water pump impeller in order to identify a parameter that can effectively determine the wear rates of the slurry pump impeller. The wear environment used was a mixture of solid particles and water. The findings revealed that erosion was the predominant type of wear observed in the experiment and the weight loss of the impeller was attributed to material removal caused by erosive wear.

Studies [11,12,13,14,15,16] investigated the type and level of mechanical stresses corresponding to the most exposed parts of the mixers, namely the blades and the arms.

Another significant challenge for the concrete mixers is the abrasive wear that can produce in both wet and dry conditions. Studies [7,15,16] were conducted to determine the stress states in the arms and mixer blades subjected to abrasive wear.

Jungedal [17] investigated the wear resistance (measuring sliding wear resistance and impact wear) of more than 30 types of steel, simulating, through an experimental stand, the concrete mixer with crushed granite mainly. The wear losses were evaluated by conducting weight measurements before, during, and after testing. A correlation was identified between hardness and wear resistance, with higher hardness levels demonstrating improved resistance to wear in the specific application under investigation. Furthermore, it was observed that the shape and degradation of abrasives had an impact on the wear rate.

The scientific literature in [18,19,20,21] primarily focused on the wear characteristics of concrete mixers, specifically analyzing the wear behavior of blades and evaluating the performance of new blade designs using a combination of numerical simulations, experimental tests, and 3D optical scanning techniques. These studies demonstrate that the new blade designs significantly reduce wear, increase durability, improve mixing efficiency, and lower maintenance costs and time.

In paper [22], an evaluation was conducted on pastes and mortars mixed for various durations and different numbers of mixer revolutions. The focus was on assessing early-age properties, such as time-dependent ion concentration in the solution, flowability, setting time, and chemical shrinkage. The findings reveal that these early-age characteristics of laboratory-mixed pastes and mortars are affected by the duration of mixing and the number of mixer revolutions. Also, Prasittisopin and Trejo [23] examined the impact of mixing time and the number of mixer revolutions on the hardened properties of laboratory-produced mortars. The results demonstrate that longer mixing times are associated with higher compressive strengths after one day but lower strengths after 28 days. Additionally, increasing the number of mixer revolutions can lead to higher porosity, resulting in reduced strength. A similar study [24] presents the findings regarding the influence of mixing time and number of mixer revolutions on the properties of laboratory-produced pastes and mortars containing 20% and 50% fly ash, demonstrating that as the mixing time and number of mixer revolutions increase, the mixtures incorporating fly ash as a replacement material exhibit enhanced fresh and hardened properties.

The research in [25] focuses on the distinct aspects of a new industrial process and its impact on engineering requirements, particularly the mechanical and microstructural characteristics of castings. The study also highlights the essential directions for further exploration of the potential of this emerging technology in producing high-quality castings. To assess the effects of ablation sand casting on the AlSi7Mg alloy impeller, investigations were conducted on various process parameters, such as binder ratio, AFS number, and pouring temperature. The evaluation includes dimensional accuracy, hardness, and ultimate tensile strength as key factors.

The equipment used in concrete production is designed to withstand the hard operational conditions imposed by the corrosive nature of the water–cement dust mixture and the abrasive properties of mineral aggregates. The active components of this equipment (especially in the case of concrete mixers) often involve costly materials and specialized manufacturing technologies. Notably, the walls, mixing blades, and arms of these mixers require special maintenance due to the fact that they are made of special materials. The maintenance activities for the entire plant are influenced by the replacement intervals of these components. Taking into account the challenges mentioned above, this research aims to provide recommendations for selecting appropriate materials for manufacturing mixing blades, based on their tribological characteristics, using both analytical methods and experimental investigations conducted on a stand designed to reproduce the mixers’ functioning conditions.

## 2. Materials and Methods

The experimental analysis focused on the constructive–functional and material characteristics of a double-shaft mixer used in the concrete manufacturing facility operated by STRABENBAU LOGISTIC SRL, Blejoi, Romania.

The experimental research was conducted to evaluate the wear behavior of the materials used in the manufacture of mixer blades, using an experimental stand to reproduce, in laboratory conditions, the concrete mixer operation.

**Figure 2:**
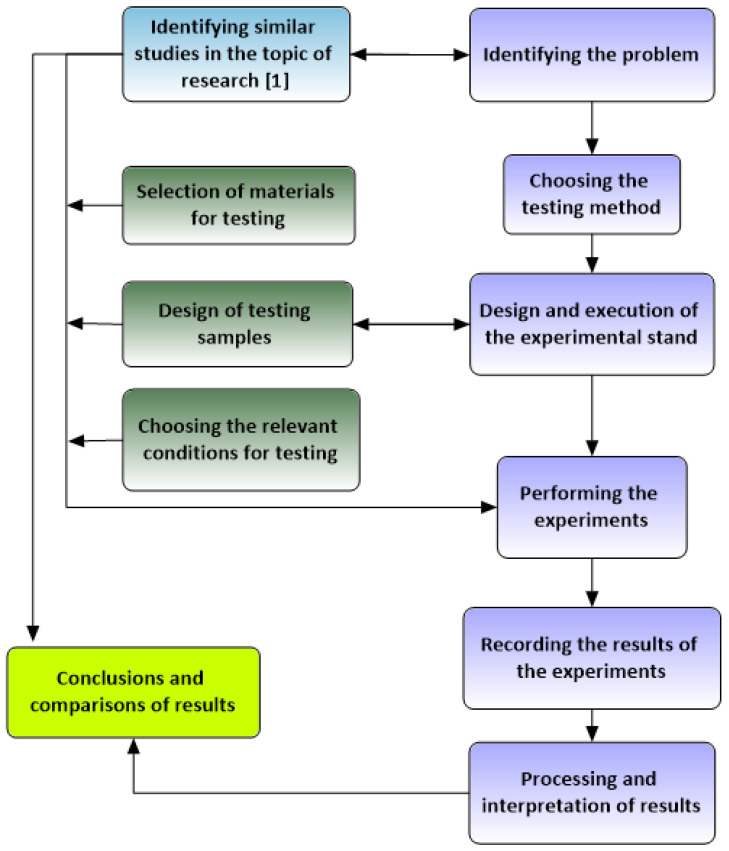
The steps of the research program.

Figure 2 shows the steps taken to perform the present study, including both experimental and analytical methods of analysis.

**Figure 3:**
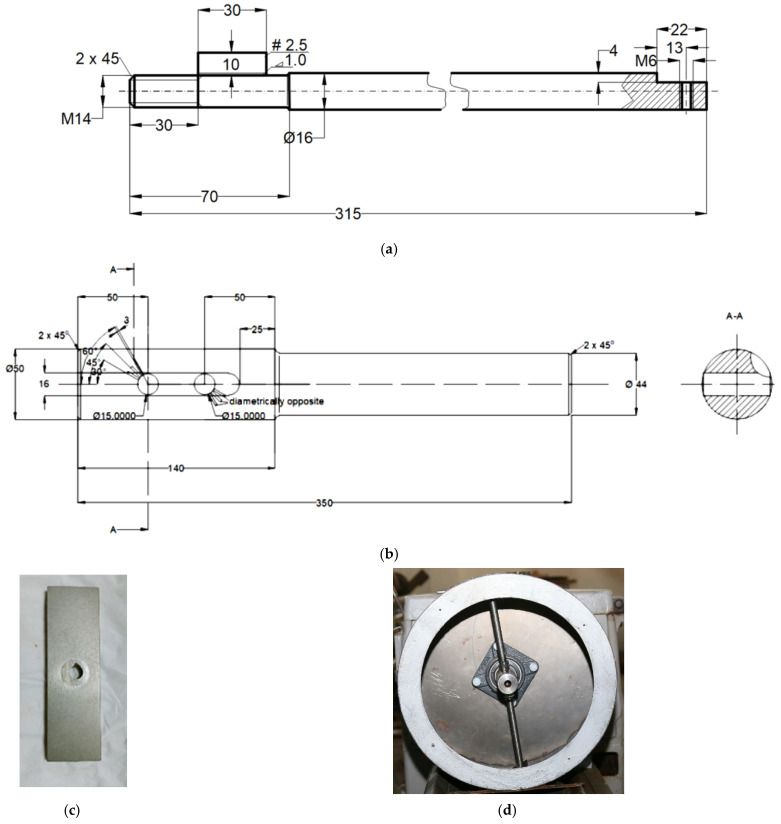
Components of the experimental stand: (**a**) drawing execution mixer arm, (**b**) drawing execution drive shaft, (**c**) sample blade, and (**d**) drum with the mentioned elements.

The experimental stand was designed to reproduce, on a scale of 1:2, the real mixer shaft in the concrete mixing plant. The stand consisted of a cylindrical bowl with a window at the top for feeding the mineral aggregates but also to allow the mixer arms to be fitted/removed. A shaft (in a bearing), with two mixing arms, was mounted inside the bowl, and the position of the shaft could be changed, relative to the shaft axis, after a fixed generator (Figure 3). At the ends, the two mixing arms were fitted with parallelepiped mixing blades (79 mm × 24 mm × 4 mm), which could be removed and replaced. This allowed the use of blades (as test samples) made of different materials.

**Figure 4:**
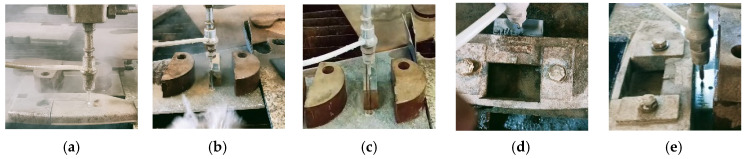
Cast-iron sample preparation on the water-jet-cutting machine: (**a**) raw sample, (**b**) sampling by thickness cutting—first face, (**c**) sampling by thickness cutting—second face, (**d**) drilling the specimen in the central area at 13 mm from the longest dimension, and (**e**) cutting at 24 mm dimension.

The cast-iron samples (the mixing blades of the experimental stand) were obtained by cutting with a water jet to avoid the thermal influence of the material characteristics (Figure 4). The faces of the blades were processed, finally, by grinding on a plane–peripheral machine.

For the tests, three types of cast-iron materials alloyed with different Cr contents (samples no. 1, no. 2, and no. 3 with, respectively, 4% Cr, 9% Cr, and 25% Cr) were chosen.

### 2.1. Chemical Composition and Hardness of the Studied Samples

Table 1 presents the chemical compositions of the three cast-iron samples, determined by the optical-emission-spectroscopy method in the Mechanical Engineering Department Laboratory at the Petroleum–Gas University of Ploiești.

The hardness characteristics of the three types of material used in the tests (hypoeutectic white cast iron) depend on the content of Cr [26,27,28,29] and are shown in Table 2.

**Figure 5:**
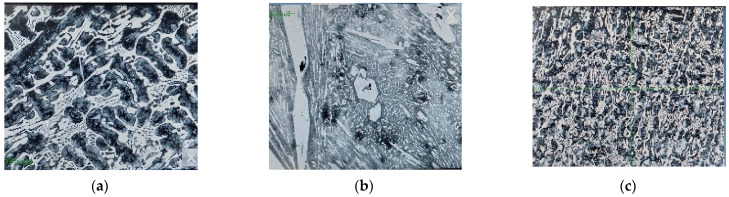
The microstructural details of the analyzed materials: (**a**) blade no. 1, (**b**) blade no. 2, and (**c**) blade no. 3.

Figure 5 presents the microstructural characteristics of the three sample materials.

It can be observed that the microstructure of the three samples presents distinctive characteristics specific to white hypoeutectic cast irons, with different forms of hard constituents, which vary based on the chromium (Cr) content.

### 2.2. Determination of Microgeometrical Parameters of the Samples

**Figure 6:**
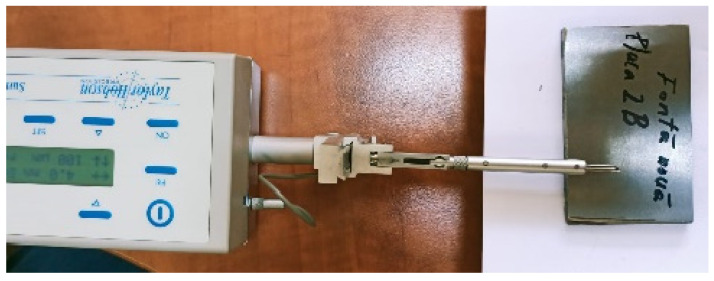
The SURTRONIC 3+ profilometer used for the measurement process.

**Figure 7:**
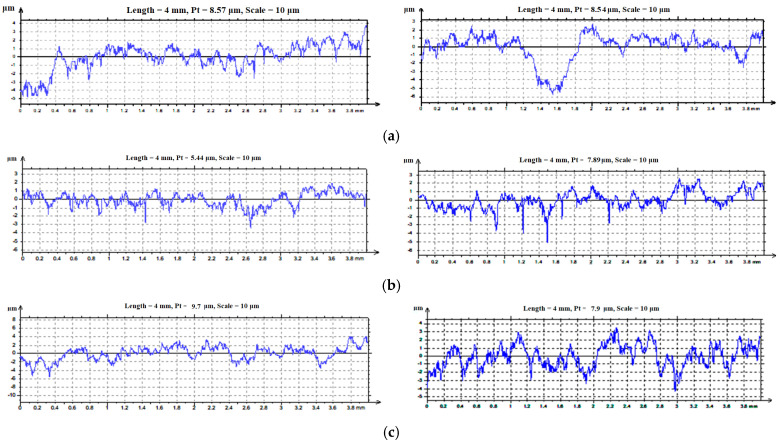
The profile curves for contact surfaces of samples: (**a**) 1A, 1B; (**b**) 2A, 2B; and (**c**) 3A, 3B.

The microgeometrical characteristics of the sample surfaces were measured with the SURTRONIC 3+ profilometer [30,31] (Figure 6) and are presented in Table 3 and Figure 7.

### 2.3. Description of the Experimental Method

The test program included, for each type of material (cast iron), 3 sets of tests consisting of subjecting the specimens to a wear process generated by the friction between the active surfaces of the blades, rotating inside the drum, and the aggregate introduced into the mixing space. In this phase of the research tests, dry aggregates were used. According to SR EN 12620-A1:2008, the dry aggregates have the following characteristics: granulometric class 4–8 mm, content of fine particles 1.5%, chloride content < 0.0025%, shape index SI 15, and water absorption WA 24.

**Figure 8:**
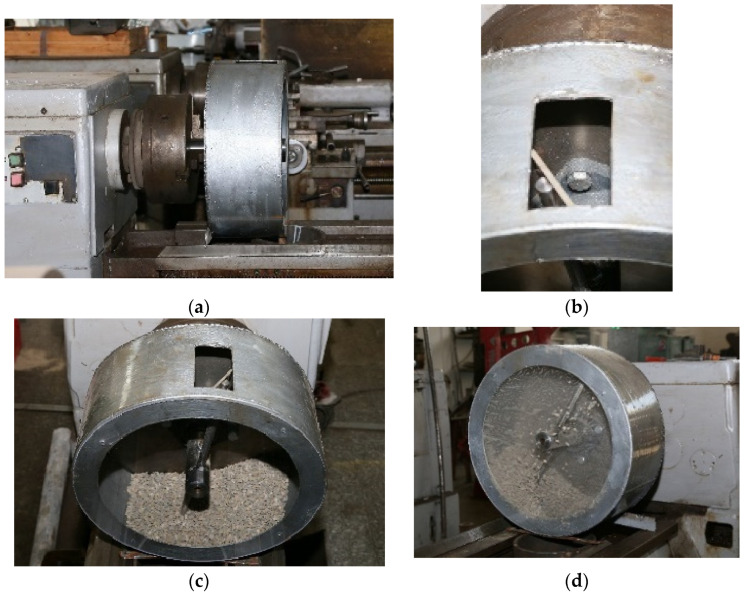
Experimental stand: (**a**) front view, (**b**) window for mounting arms, (**c**) 5 kg of mineral aggregates, and (**d**) rotational motion.

The rotation of the mixing arms was determined by the main motor of a universal lathe, type SN 400. The entire experimental stand was mounted on the normal lathe (Figure 8).

The wear of the cast-iron samples (blades) was determined gravimetrically by weighing them at different time intervals using an analytical balance, type KERN ALJ, with an accuracy of 10^−4^ g.

Before each weighing, the cast-iron samples were prepared by washing with methyl ethyl ketone (MEK), wiping with a solvent-resistant microfiber cloth, and drying in warm air. For good reproducibility of the tests, the quantity of aggregates was replaced before each cycle, respecting the particle-size class and the mass of mineral material. This ensured the same testing environment conditions for each stage of the experimental program.

In order to characterize as fully as possible the behavior of the studied materials under operating conditions, the experimental program involved three sets of tests, for each type of material, characterized by different angles of inclination of the active face of the blade relative to the shaft axis (30, 45, and 60 degrees).

The aggregate material used in the tests was crushed stone with a grain size of 4–8 mm. The quantity of stone used in each test cycle was 5 kg. The rotating speed of the main shaft during all tests was 40 rpm.

### 2.4. Theoretical Method Used to Confirm the Experimental Results

**Figure 9:**
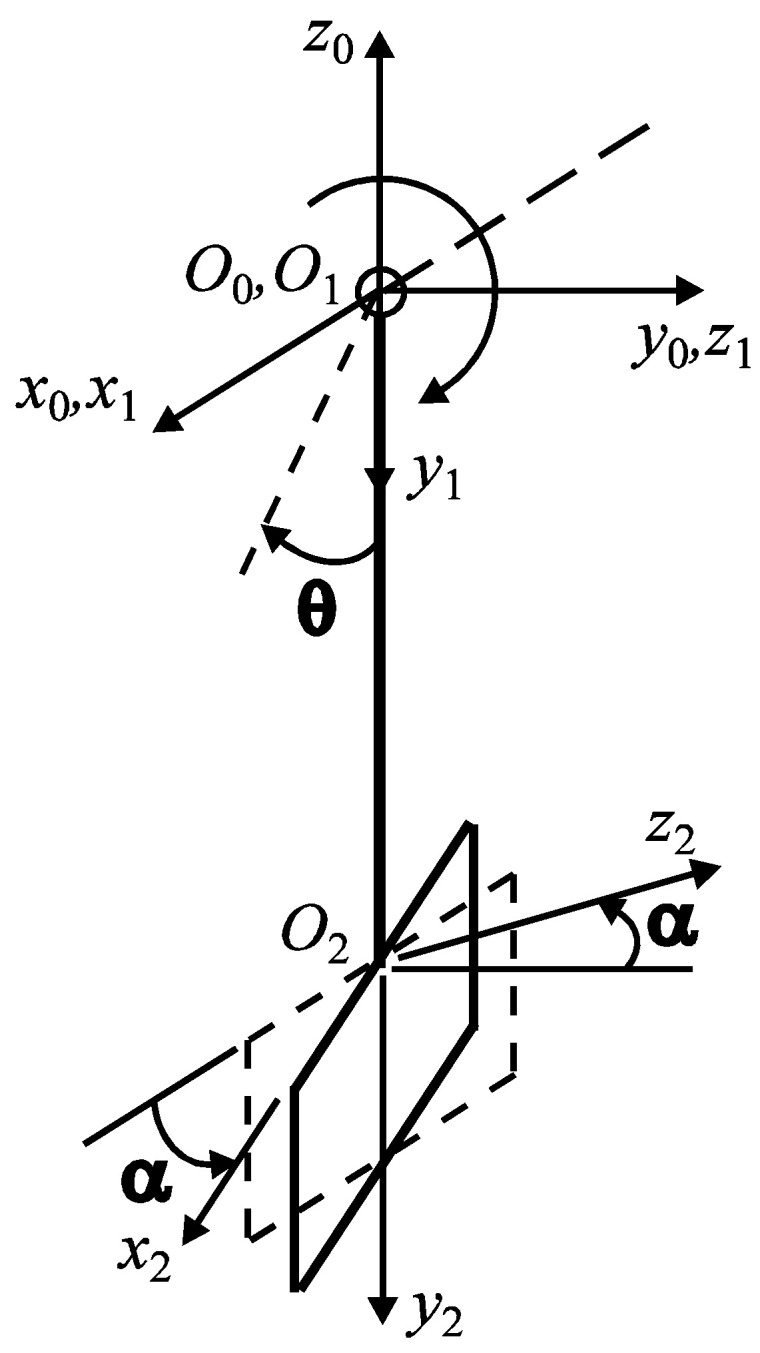
Schematic representation of the mixing arm and the blade.

In order to evaluate, exclusively, the effect of the friction of the aggregate particles on the mixing blades, an analytical model was created. Therefore, this research studied the influence on the ratio between the frictional force (manifested on the surface of the mixing blade) and the weight of the aggregate material from the mixer bowl—of the angle of inclination of the active face of the blade, relative to the shaft axis α—as presented in Figure 9.

The frictional force $F_{f}$ is determined with the relation:

(1) $$F_{f}=\mu \cdot N$$

where μ is the sliding frictional coefficient between the aggregate material and the blade and *N* is the normal reaction to the blade surface, corresponding to its loading with the aggregate material.

If we denote by *G* the weight of the aggregate material that loads the blade, then $N=G_{z_{2}}$, where $G_{z_{2}}$ is the component of the weight *G* along the axis $O_{2}z_{2}$.

The projections of the weight *G* on the axes of the coordinate system $O_{2}x_{2}y_{2}z_{2}$, grouped in the vector $G^{\left(2\right)}$, are determined with the relation

(2) G(2)=R02⋅G(0),

where $G^{\left(0\right)}$ is the vector containing the projections of the weight *G* on the axes of the fixed coordinate system $O_{0}x_{0}y_{0}z_{0}$($G^{\left(0\right)}={\left[\begin{array}{ccc} 0 & 0 & -G \end{array}\right]}^{T}$) and R02 is the rotation matrix corresponding to the relative orientation between the coordinate systems $O_{2}x_{2}y_{2}z_{2}$ and $O_{0}x_{0}y_{0}z_{0}$ during the operation

(3) R02=R2T0,

(4) R20=R10⋅R21,

where, as can be seen in Figure 9,

(5) R10=R(x,−(θ+π/2)) R21=R(y,−α)

## 3. Results

### 3.1. Experimental Method

**Figure 10:**
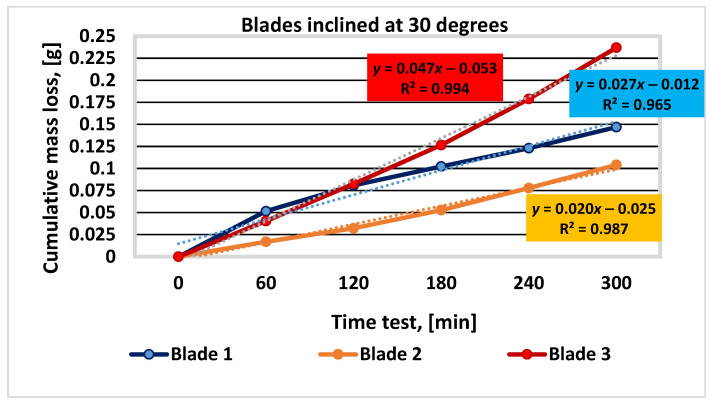
Graphical representation of the experimental results for blades inclined at 30 degrees.

**Figure 11:**
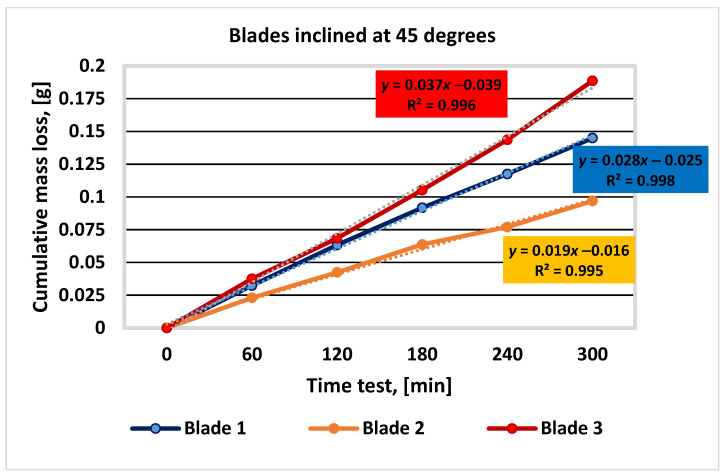
Graphical representation of the experimental results for blades inclined at 45 degrees.

**Figure 12:**
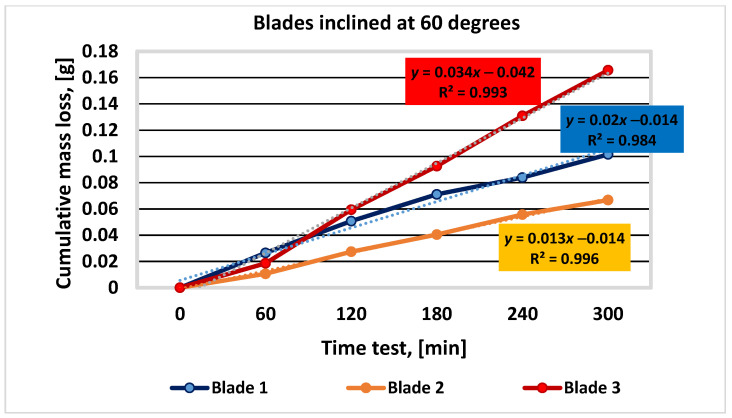
Graphical representation of the experimental results for blades inclined at 60 degrees.

The results of the three sets of tests, corresponding to the blade tilt angles of 30, 45, and 60 degrees, for each of the three materials analyzed, are shown in Table 4, Table 5 and Table 6 and Figure 10, Figure 11 and Figure 12.

**Figure 13:**
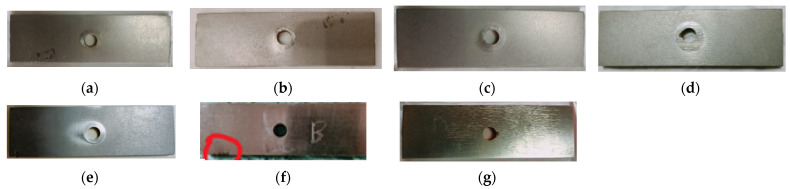
The selective aspects of the samples after different test periods (noting that when operating duration is increased, the traces of abrasive–erosive wear become more pronounced): (**a**) sample blade 1, after 60 min test, inclination 30 degrees; (**b**) sample blade 1, after 120 min test, inclination 30 degrees; (**c**) sample blade 2, after 300 min test, inclination 30 degrees; (**d**) sample blade 3, after 240 min test, inclination 30 degrees; (**e**) sample blade 1, after 300 min test, inclination 60 degrees; (**f**) sample blade 3, after 60 min test, inclination 60 degrees (noting sample chipped due to entrapment of an aggregate grain between the blade and the drum); and (**g**) sample blade 3, after 300 min test, inclination 60 degrees.

The selective aspect of the samples after different testing periods can be seen in Figure 13.

**Table 4:** Mass loss values determined during the experimental test for blades inclined at 30 degrees.

Duration of Operation, (min)	Sample Mass during Experiment; Mass Loss; Δ*m*; Respectively, ΣΔ*m*; (g)					
	Blade 1		Blade 2		Blade 3	
0	55.5200	0	54.7568	0	70.4701	0
60	(Figure 13a) 55.4684	0.0516	54.7399	0.0169	70.4297	0.0404
	0.0516		0.0169		0.0404	
120	(Figure 13b) 55.4392	0.0808	54.7246	0.0322	70.3876	0.0825
	0.0292		0.0153		0.0421	
180	55.4177	0.1023	54.7040	0.0528	70.3435	0.1266
	0.0215		0.0206		0.0441	
240	55.3970	0.123	54.6790	0.0778	70.2913	0.1788
	0.0207		0.0250		(Figure 13d) 0.0522	
300	55.3731	0.1469	(Figure 13c) 54.6527	0.1041	70.2330	0.2371
	0.0239		0.0263		0.0583

**Table 5:** Mass loss values determined during the experimental test for blades inclined at 45 degrees.

Duration of Operation, (min)	Sample Mass during Experiment; Mass Loss; Δ*m*; Respectively, ΣΔ*m*; (g)					
	Blade 1		Blade 2		Blade 3	
0	55.3731	0	53.3737	0	69.7735	0
60	55.3408	0.0323	53.3507	0.0230	69.7360	0.0375
	0.0323		0.0230		0.0375	
120	55.3098	0.0633	53.3313	0.0424	69.7051	0.0684
	0.0310		0.0194		0.0309	
180	55.2814	0.0917	53.3100	0.0637	69.6683	0.1052
	0.0284		0.0213		0.0368	
240	55.2556	0.1175	53.2967	0.077	69.6299	0.1436
	0.0258		0.0133		0.0384	
300	55.2282	0.1449	53.2768	0.0969	69.5849	0.1886
	0.0274		0.0199		0.0450

**Table 6:** Test results on the experimental stand for blades inclined at 60 degrees.

Duration of Operation, (min)	Sample Mass during Experiment; Mass Loss; Δ*m*; Respectively, ΣΔ*m*; (g)					
	Blade 1		Blade 2		Blade 3	
0	54.3974	0	54.6199	0	70.2322	0
60	54.3709	0.0265	54.6093	0.0106	70.1795	0.0186
	0.0265		0.0106		(Figure 13f) 0.0527 * 0.0186	
120	54.3467	0.0507	54.5925	0.0274	70.1387	0.0594
	0.0242		0.0168		0.0408	
180	54.3263	0.0711	54.5794	0.0405	70.1055	0.0926
	0.0204		0.0131		0.0332	
240	54.3135	0.0839	54.5642	0.0557	70.0672	0.1309
	0.0128		0.0152		0.0383	
300	(Figure 13e) 54.2958	0.1016	54.5531	0.0668	70.0324	0.1657
	0.0177		0.0111		(Figure 13g) 0.0348	

* The value was corrected by reduction with the value of the chip mass (0.0341 g) resulting from the blocking of a grain of aggregate between the mixing blade and the drum wall. The chip mass was determined indirectly by using a silicone moulage, corresponding to the void remaining in the body of the mixing blade after detachment. By weighing the blade—after the test (with the gap after chip removal) and after filling with the silicone molding—the mass of the molding was determined (70.1853 − 70.1795 = 0.0058 g). The mass of the detached chip was calculated with reference to the density values of the two materials, cast iron (7.6 kg/dm^3^) and silicone (1.2937 kg/dm^3^, determined by dipping a silicone sphere of 10 g in mass into a graduated cylinder).

### 3.2. Theoretical Method

**Figure 14:**
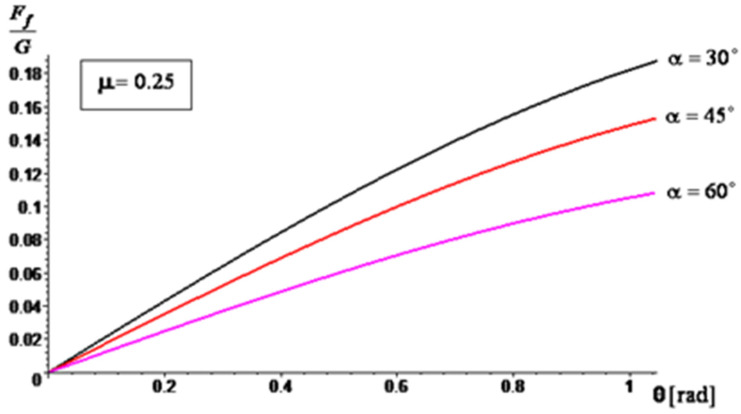
The ratio variation, $F_{f}/G$ for μ=0.25.

**Figure 15:**
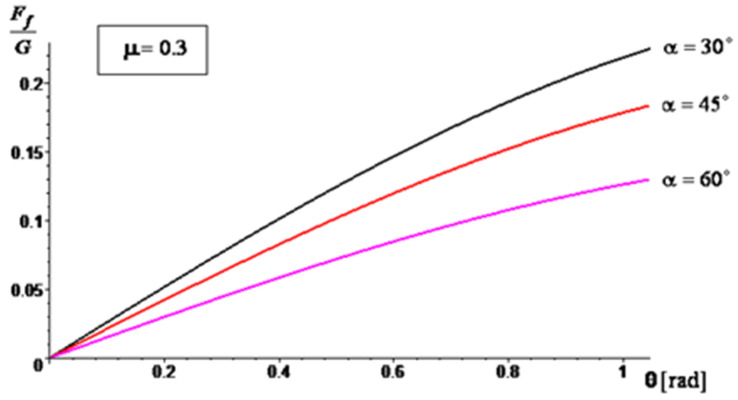
The ratio variation, $F_{f}/G$ for μ=0.30.

**Figure 16:**
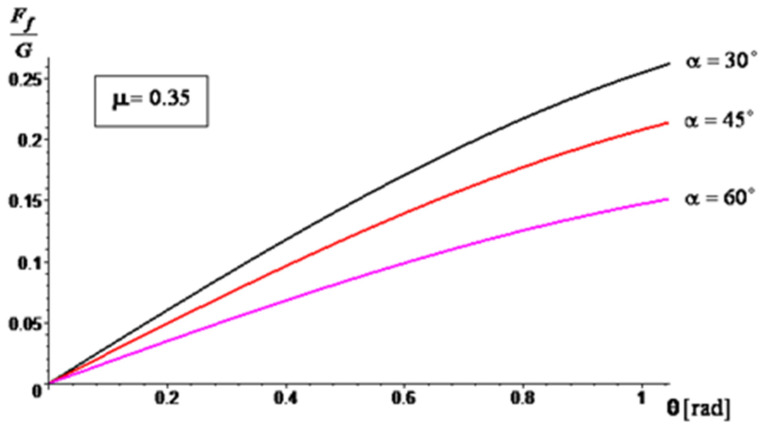
The ratio variation, $F_{f}/G$ for μ=0.35.

A series of simulation results are presented in Figure 14, Figure 15 and Figure 16 regarding the variation of the ratio between the frictional force (manifested on the surface of the mixing blade) and the weight of the aggregate material that loads the blade ($F_{f}/G$) during operation, depending on the angle θ. For the angle of inclination α of the active face of the blade, relative to the shaft axis, three cases were considered: $\alpha =30^{\circ }$, $\alpha =45^{\circ }$ and $\alpha =60^{\circ }$. For the sliding frictional coefficient μ between the aggregate material and the blade, the following cases were considered: μ = 0.25, μ = 0.3, and μ = 0.35. The simulations were performed for θ∈[0,π/3].

## 4. Discussion

After analyzing the results presented in Table 4, Table 5 and Table 6 and Figure 9, Figure 10 and Figure 11, it can be concluded that blade no. 2 (with 26% Cr) has the smallest mass loss (smaller with 50%, … % and 90%, … 127% compared to blade no. 1 (4% Cr) and blade no. 3 (9% Cr), respectively) and therefore the best wear resistance for all the values of inclination angle of the blade. These conclusions are similar to those from scientific paper [1], where the authors investigated the wear behavior of the same materials but using a different experimental method.

Blade no. 1 has a better wear behavior than blade no. 3. The differences between the cumulative mass loss between blades no. 1 and no. 3 are 61.4% (for the inclination angle of 30 degrees), 30.16% (for the inclination angle of 45 degrees), and 63.09% (for the inclination angle of 60 degrees).

On the other hand, when the inclination angle of the blade relative to the shaft axis increases, the cumulative mass loss decreases, with the maximum values of 44.58% for blade no. 1, 55.83% for blade no. 2, and 43% for blade no. 3.

Numerous striations/rhizomes (resulting from the abrasive-–erosive action of the aggregates) were observed on the sample surfaces (Figure 13).

The results of the simulations show that by increasing the angle α, the ratio $F_{f}/G$ decreases. This decrease is more accentuated when the sliding frictional coefficient between the aggregate material and the blade increases. Therefore, as the angle of inclination increases, the frictional force experienced by the blade decreases in relation to the weight of the aggregate material being carried by the blade. This suggests that a higher angle of inclination can potentially reduce the load and frictional forces on the blade during operation, which may have implications for the performance and wear characteristics of the mixer. The analytical method through the obtained results confirms the experimental results, so the testing method proposed by the authors can be used to also assess the wear behavior of other similar industrial equipment.

## 5. Conclusions

This paper presents the results of tests on corrosive–abrasive wear obtained with an experimental stand designed and built by the authors with reference to a real concrete mixer. The stand was capable of reproducing in-laboratory conditions, the phenomena that occur during the operation of real concrete mixers, related to the loadings of an abrasive–erosive nature to which the mixing blades are subjected.

Through the proposed experimental program, the authors wanted to verify the conformity of the obtained results using this method (in approximately similar conditions as industry) with the results obtained through previous tests [1], where laboratory instruments specific to the tribological tests of materials were used (the Baroid-type tribometer).

In order to justify the mechanical phenomena, the paper also presented an algorithm for calculating the frictional forces that develop when the aggregate particles move on the surface of the mixing blades during the mixer operation.

The main conclusions of the paper are as follows:

✓ The analytical calculation regarding the influence of the inclination angle and the sliding frictional coefficient between the aggregate material and the blade—on the ratio between the frictional force (manifested on the surface of the mixing blade) and the weight of the aggregate material that loads the blade during operation—suggests that modifying the inclination angle can have a significant impact on the frictional forces experienced by the mixing blade during mixing;
✓ When the angle of inclination is increased, the relative contribution of frictional forces to the overall load on the mixing blade decreases, indicating a potential reduction in wear and mechanical stresses on the mixer components. Moreover, the effect is more substantial when the sliding frictional coefficient between the aggregate material and the mixing blade is higher, emphasizing the importance of considering material properties and surface interactions in mixer design and operation. Overall, these results highlight the potential benefits of optimizing the angle of inclination in mixer blade design to minimize frictional forces and improve the longevity and performance of the mixer in handling aggregate materials with varying frictional characteristics;
✓ The experimental results (graphically represented in Figure 10, Figure 11 and Figure 12) confirm and are in full agreement with the observations reported in the paper [1], which presents a wear test method using the Baroid tribometer. According to these results, mixing blade no. 2 with 26% Cr exhibits the best wear resistance among the tested mixing blades, while mixing blade no. 1 (with 4% Cr) performs better than mixing blade no. 3 (with 9% Cr). These conclusions are consistent with the results mentioned in paper [1]. In addition, the results specified in this paper demonstrate that this behavior of the materials is maintained under the conditions of different inclination angles of the mixing blades. This conclusion superimposed on the effect of inclination angle can provide important information for choosing the best technical solution for the concrete-mixer design;
✓ Regarding the chemical composition of the mixing-blade materials, it can be stated (in accordance with the assessments from [1]) that the chromium content plays a significant role in determining the wear behavior of the three types of cast iron;
✓ According to reference [26], cast irons with chromium percentages above 10% show increased wear resistance. The typical range of Cr content assumed in the formulas for such cast irons is 10–35%, being in accordance with the experimental results obtained for mixing blade no. 2;
✓ In industrial applications related to the production of cement concrete, it is advisable to use cast iron with either 25% Cr or 4% Cr. The choice of the intermediate grade (9% Cr) is not justified due to the higher cost and inferior performance compared to the 4% Cr version. Detailed explanations are given in [1];
✓ Additionally, the design and construction aspects demonstrating (in accordance with the experimental results) that increasing the angle of inclination of the mixing blades relative to the shaft axis leads to decreased cumulative mass loss and improved wear resistance for all mixing blades must be considered;
✓ As the angle of inclination of the mixing blades increases, erosive wear becomes predominant, accelerating the phenomenon of mechanical degradation of the samples. When choosing the right angle of inclination of the mixing blades, the mixing efficiency ensured by the position of the mixing blades must also be taken into account [14]. In this way, by using the observations related to the materials used for the mixing blades, the angle of inclination of the mixing blades, and the efficiency of the mixing process, optimal solutions can be found for the design and manufacture of concrete mixers;
✓ The method proposed by the authors can be recommended for the study of wear phenomena in similar industrial equipment.

## Figures and Tables

**Table 1:** The chemical composition of cast-iron samples.

Sample	Chemical Composition, %								
	C	Si	P	S	Cr	Mn	Fe	Ni	Mo
**1**	3.28	0.76	0.07	0.03	3.83	1.10	89.86	0.89	0.20
**2**	3.72	0.74	0.02	0.04	25.65	0.87	68.19	0.040	0.35
**3**	3.08	0.96	0.04	0.03	9.77	1.14	84.12	0.35	0.49

**Table 2:** Hardness values for samples subjected to wear tests at the beginning and end of the test program.

Value of Tilt-Angle Blade Relative to the Axis of Rotation, (Degrees)	Blade—Cast Iron 1 HV0.2		Blade—Cast Iron 2 HV0.2		Blade—Cast Iron 3 HV0.2	
	Unworn Sample	Worn Sample	Unworn Sample	Worn Sample	Unworn Sample	Worn Sample
30	464.654, value from [1]	1191	1228.505, value from [1]	911	632.9904, value from [1]	676.5
45		1237		1029		936.5
60		1320		1260.5		1177

**Table 3:** Roughness values of cast-iron samples.

Sample/Sample Face	1A	1B	2A	2B	3A	3B
**Roughness value** ***R_a_*, μm**	0.555	0.569	0.452	0.528	0.810	0.805

## Data Availability

Data are contained within this article.
